# Abiotic stress and genome dynamics: specific genes and transposable elements response to iron excess in rice

**DOI:** 10.1186/s12284-015-0045-6

**Published:** 2015-02-25

**Authors:** Taciane Finatto, Antonio Costa de Oliveira, Cristian Chaparro, Luciano C da Maia, Daniel R Farias, Leomar G Woyann, Claudete C Mistura, Adriana P Soares-Bresolin, Christel Llauro, Olivier Panaud, Nathalie Picault

**Affiliations:** Plant Genomics and Breeding Center, Eliseu Maciel School of Agronomy, Federal University of Pelotas, 96010-610 Pelotas, RS Brazil; Laboratoire Génome et Développement des Plantes, UMR 5096, Université de Perpignan Via Domitia, F-66860 Perpignan, France; CNRS, Laboratoire Génome et Développement des Plantes, UMR 5096, F-66860 Perpignan, France; Present address: Universidade Tecnológica Federal do Paraná, Campus Pato Branco, 85503-390 Pato Branco, PR Brazil; Present address: Laboratoire Ecologie et Evolution des Interactions, UMR 5244, F-66860, Université de Perpignan Via Domitia, Perpignan, France

**Keywords:** Rice, Microarray, Iron toxicity, LTR-retrotransposon, *cis*-regulatory elements

## Abstract

**Background:**

Iron toxicity is a root related abiotic stress, occurring frequently in flooded soils. It can affect the yield of rice in lowland production systems. This toxicity is associated with high concentrations of reduced iron (Fe^2+^) in the soil solution. Although the first interface of the element is in the roots, the consequences of an excessive uptake can be observed in several rice tissues. In an original attempt to find both genes and transposable elements involved in the response to an iron toxicity stress, we used a microarray approach to study the transcriptional responses of rice leaves of cv. Nipponbare (*Oryza sativa* L. ssp. *japonica*) to iron excess in nutrient solution.

**Results:**

A large number of genes were significantly up- or down-regulated in leaves under the treatment. We analyzed the gene ontology and metabolic pathways of genes involved in the response to this stress and the *cis*-regulatory elements (CREs) present in the promoter region of up-regulated genes. The majority of genes act in the pathways of lipid metabolic process, carbohydrate metabolism, biosynthesis of secondary metabolites and plant hormones. We also found genes involved in iron acquisition and mobilization, transport of cations and regulatory mechanisms for iron responses, and in oxidative stress and reactive oxygen species detoxification. Promoter regions of 27% of genes up-regulated present at least one significant occurrence of an ABA-responsive CRE. Furthermore, and for the first time, we were able to show that iron stress triggers the up-regulation of many LTR-retrotransposons. We have established a complete inventory of transposable elements transcriptionally activated under iron excess and the CREs which are present in their LTRs.

**Conclusion:**

The short-term response of Nipponbare seedlings to iron excess, includes activation of genes involved in iron homeostasis, in particular transporters, transcription factors and ROS detoxification in the leaves, but also many transposable elements. Our data led to the identification of CREs which are associated with both genes and LTR-retrotransposons up-regulated under iron excess. Our results strengthen the idea that LTR-retrotransposons participate in the transcriptional response to stress and could thus confer an adaptive advantage for the plant.

**Electronic supplementary material:**

The online version of this article (doi:10.1186/s12284-015-0045-6) contains supplementary material, which is available to authorized users.

## Background

Rice, *Oryza sativa* L., is the staple food for more than two thirds of the world’s population and is the second most widely grown cereal. Beyond its social and economic importance, rice is a model plant among the monocots because it has a small genome (~389 Mbp), which has been sequenced for the cultivars Nipponbare (*japonica* rice) (International Rice Genome Sequencing Project 2005) and 93–11 (*indica* rice) (Yu et al. 2002). Iron (Fe) is essential to mineral nutrition of plants. It is necessary for photosynthesis, electron transport and other redox reactions (Marschner 1995). Fe is subjected to tight control to avoid cellular toxicity (Connolly and Guerinot 2002). Although essential, it can be toxic when in excess (Stein et al. 2009a). Iron toxicity is one of the most important constraints to rice production on acid soils. The uptake of excessive Fe(II) by rice roots and its xylem transport *via* the transpiration stream into the leaves can lead to the generation of reactive oxygen species (Thongbai and Goodman 2000), causing the typical leaf-bronzing symptoms and entailing yield losses in the range of 10%-100% (Audebert and Fofana 2009).

Higher plants have evolved two main strategies for Fe acquisition from the rhizosphere: Fe reduction (Strategy I) or Fe chelation (Strategy II). Strategy I is employed by all plant species, with the exception of graminaceous plants, and involves pumping protons by H^+^-ATPases to acidify the rhizosphere and increase Fe solubility in the soil. A ferric chelate reductase (FRO) reduces Fe^3+^ to Fe^2+^, and Fe^2+^ transporters (IRTs - Iron Reduced Transporters) carry Fe into cells (Kim and Guerinot 2007). The Strategy II Fe-uptake system is limited to graminaceous plants, which release mugineic acid family phytosiderophores (MAs) from their roots to solubilize the sparingly soluble Fe^3+^ in the soil (Takagi 1976; Takagi et al. 1984; Ishimaru et al. 2006). Exceptionally, in addition to a chelation strategy, rice possesses an Fe-uptake system that directly absorbs Fe^2+^ (strategy I) (Ishimaru et al. 2006). This is advantageous for growth in submerged conditions because, unlike other grasses, rice is well adapted to grow in flooded conditions where Fe^2+^ is more abundant than Fe^3+^ (Ishimaru et al. 2006).

Rice plants have developed morphological and physiological mechanisms for avoiding adverse iron-toxic soil conditions and large amounts of iron in the plant (Becker and Asch 2005) and/or tolerance mechanisms to cope with and survive such conditions. Three major types of adaptation strategies have been proposed by Becker and Asch 2005. Firstly, an Fe-exclusion/avoidance strategy where the plants exclude Fe^2+^ at the root level and hence avoid Fe^2+^ damage to the shoot tissue (rhizospheric oxidation and root ion selectivity). Secondly, the Fe-inclusion/avoidance strategy where Fe^2+^ is taken up into the rice root, but tissue damage may be avoided by either compartmentalization (immobilization of active iron in “dumping sites”, *e.g.*, old leaves or photosynthetically less active leaf sheath tissue) or exclusion from the symplast (immobilization in the leaf apoplast). Finally, the Fe-inclusion/tolerance strategy where plants tolerate elevated levels of Fe^2+^ within leaf cells, probably *via* enzymatic “detoxification” in the symplast (Becker and Asch 2005; Stein et al. 2009a). However, at present, these mechanisms are not very well characterized.

Several studies aiming to identify genes involved in response of rice to iron have been described, although most published studies concern iron deficiency (Kobayashi and Nishizawa 2012). Iron homeostasis has mainly been studied through differential expression of target genes, for example genes related to iron transport such as *OsIRT* (Lee and An 2009), *OsNRAMP1 and OsNRAMP2* (Zhou and Yang 2004), storage proteins like ferritin (*OsFER*) (Silveira et al. 2009; Stein et al. 2009b) and transcription factor like *OsWRKY8*0 (Ricachenevsky et al. 2010). In fact, several transporters potentially involved in metal ion homeostasis have been identified in the rice genome (Kobayashi and Nishizawa 2012). Most of these metal transporters are capable of transporting one or several divalent cations including Fe^2+^, Zn^2+^, Mn^2+^ and Cu^2+^ (Narayanan et al. 2007). However, the contribution of each transporter and the precise iron flux still need to be clarified for each step involved in iron translocation.

In addition, the responses of plants to stress are complex and its perception requires interaction between multiple sensors. After initial recognition of stress by cells, a signal transduction cascade is triggered through secondary messengers that transmit the signal, activating responsive genes and generating an initial response. The products of the induced genes may be involved in response to stress and signal transduction. The stress genes enable plants to support these adverse conditions through short and long term responses (Grennan 2006). Transcription factors (TFs) regulate the first step of gene expression and are usually defined as proteins containing a DNA-binding domain that recognize specific DNA sequences, *cis*-acting regulatory elements (CREs), located in gene promoter regions (Mitsuda and Ohme-Takagi 2009). In order to understand mechanisms controlling gene expression in response to iron excess, it is important to know if specific CREs are present in the promoters of differentially-expressed genes.

The rice genome harbors a significant proportion of transposable element-related sequences (at least 33%) (IRGSP 2005). The most predominant type of transposable elements (TEs) are long terminal repeat (LTR) retrotransposons, with nearly 400 families ranging from one to several hundred copies, which constitute over 90 Mbp of the genome (*i.e.* 23%) (El Baidouri and Panaud 2013). However, the general impact of TEs on the structure, evolution and function of plant genomes are not yet fully understood (Ma et al. 2004; Bennetzen 2007). LTR-retrotransposons require transcription and translation of the genetic information they encode in order to be transpositionally active. In fact, class I elements transpose *via* their mRNA, which is synthesized by RNA polymerase II (Kumar and Bennetzen 1999). The existence of transpositionally active LTR-retrotransposons in rice has only been demonstrated for 11 elements (Hirochika et al. 1996; Picault et al. 2009; Wang et al. 2009; Sabot et al. 2011). Although most TEs are transpositionally inactive, they can sometimes be activated by stress (Grandbastien 1998), although the exact process of transcription induction remains unknown. Transcriptional activation of LTR-retrotransposons can also significantly alter the expression of adjacent genes like in the case of blood orange (Butelli et al. 2012).

Recently, a transcriptomic approach was used (Quinet et al. 2012) and revealed modifications in gene expression after 3 days of exposure to iron excess concerning genes involved in hormonal signaling but also those involved in C-compound and carbohydrate metabolism, oxygen and electron transfer, oxidative stress, and iron homeostasis and transport in *indica* rice (cv. Kong Pao). This cultivar was classified as tolerant to iron toxicity by Engel et al. (2012) whereas Nipponbare cultivar was classified as susceptible. Thus, we decided to observe if the response to iron toxicity is a feature that could depend on genotypes presenting different degrees of tolerance/susceptibility. In addition, as we have the complete sequence of the Nipponbare cultivar, we designed a microarray that contained oligomers corresponding to all genes and LTR-retrotransposons of Nipponbare with the aim of discovering the mechanisms involved in the global response of rice in iron excess condition. In the present study, we performed a complete analysis of the expression of genes and LTR-retrotransposons, identifying the metabolic pathways and genes involved in response to this stress. Furthermore, we analyzed CREs in LTR of LTR-retrotransposons and gene promoters to potentially highlight common mechanisms in response to an abiotic stress.

## Results

### Phenotyping of rice plants

**Figure 1:**
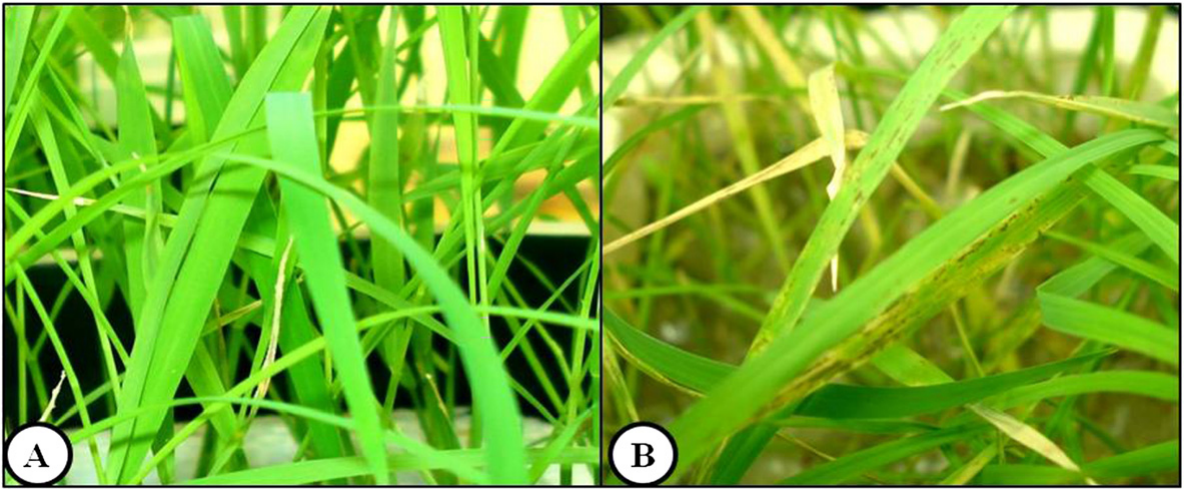
**Leaves of 18-day-old rice seedlings (** ***Oryza sativa*** **L. ssp.** ***japonica*** **cv. Nipponbare). A)** after development in a complete nutrient solution (control treatment, 10 μM Fe-EDTA) or **B)** after four days of iron excess exposure (7000 μM Fe-EDTA).

After four days of exposure to iron excess in nutrient solution, 18-day-old stressed rice plants showed necrotic spots on the leaves, which is a typical symptom of direct toxicity due to the accumulation of iron in the leaves (Figure 1). In the plants that grew in the control condition (without iron excess) no symptoms were observed and the leaves showed a uniform green color. In addition, no difference was observed in the fresh and dry matter between the plants with or without exposure to iron excess.

### Quantification of micronutrients in the tissue of rice leaves

The quantification of micronutrients was realized in shoots and significant differences (p = 0.00374) were found between iron content in leaves of 18-day-old rice seedlings after four days of iron excess exposure compared with control plants (Table 1). These results indicate that leaves of cv. Nipponbare cultivated in iron excess solution absorbed more than twice as much iron as seedlings in control conditions (optimal amount of iron). In contrast, for micronutrients such as manganese, copper and zinc, no significant differences (p > 0.05) between control and iron excess conditions were observed, indicating that the excess of iron had no influence on absorption of these cations in our conditions.

**Table 1:** **Means of micronutrient content in leaves, plant height and root length and shoot and dry matter in 18-day-old rice seedlings (** ***Oryza sativa*** **ssp.** ***japonica*** **cv. Nipponbare) after four days of iron excess exposure**

**Condition**	**Micronutrients content (mg kg** ^**−1**^ **)**				**Plant height (cm)**	**Root lenght (cm)**	**Shoot dry matter (g)**	**Root dry matter (g)**
	**Manganese**	**Cooper**	**Zinc**	**Iron**				
Control	500.50	27.72	91.68	795.75*	19.5	14.2	0.086	0.042
Iron toxicity	527.87	26.38	105.04	1927.12	18.8	13.8	0.092	0.039

*Indicate significant differences in means between control and iron toxicity conditions by ANOVA F-test.

### Differentially expressed genes in microarray

Transcriptomic profiles were obtained using microarrays on Nipponbare rice plants under iron excess condition compared to non-stressed condition (three independent biological replicates for each condition). Genes significantly regulated were selected using a *P*_adjusted_-value threshold of 0.05 and a fold change cutoff of 2 (|Log_2_(FC)| ≥ 1). A total of 2,525 genes are differentially expressed in rice under iron excess, which represent about 5.5% of the entire set of rice genes. Among these, 2,457 were up-regulated and 68 down-regulated (Additional file 1: Table S1). Thus, in our experimental conditions, more than 97% of differentially expressed genes are up-regulated.

### Microarray validation by RT-qPCR

Microarray data were confirmed by analyzing expression levels of 17 representative genes using reverse transcription quantitative polymerase chain reaction (RT-qPCR). The correlation coefficient between microarray and RT-qPCR data was 0.7856 (P < 0.01), which can be considered as a good positive correlation. Table 2 shows the relative quantification of gene expression obtained by RT-qPCR, and the gene expression (fold-change) obtained by microarray for 17 genes. These results show that the array data are in accordance with the RT-qPCR data.

**Table 2:** **Differential expression of genes in the microarray (Log**_**2**_ **FC) and relative quantification (RQ expressed in Log**_**2**_ **) by RT-qPCR in leaves of 18-day-old rice seedlings after four days of iron excess exposure**

***Locus***	**Log**_**2**_ **FC**	**RQ (log**_**2**_ **)**	**Description**
*Os02g0121700*	2.14	2.3	Terpenoid synthase domain containing protein
*Os02g0594800*	2.48	3.0	OsNAC50, No apical meristem (NAM) protein
*Os02g0740700*	1.68	2.5	Peptidase M10A and M12B
*Os05g0162000*	2.25	1.5	Similar to Peroxidase
*Os06g0257450*	2.26	3.7	Similar to Ribonucleoside-diphosphate reductase
*Os08g0467400*	1.87	0.8	OsZIP14, Zinc/iron permease family protein.
*Os08g0508000*	2.58	2.0	Cytochrome P450 family proten
*Os12g0601800*	1.67	1.2	OsbZIP88, Similar to BZIP transcription factor family
*Os06g0141200*	−2.54	−2.0	OsZFP1, Similar to RNA-binding protein EWS.
*Os05g0506000*	−1.02	−1.8	Similar to MS5-like protein
*Os10g0521900*	−1.21	−1.6	Similar to Membrane protein
*Os06g0649000*	1.41	2.5	OsWRKY28
*Os12g0567800*	2.01	1.2	OsMIT1f, Plant metallothionein, family 15 protein.
*Os12g0567800*	2.01	0.5	OsMIT1f, Plant metallothionein, family 15 protein.
*Os03g0288000*	1.12	2.0	OsMIT1b, Similar to Metallothionein
*Os05g0399300*	3.29	3.0	OsChi1b, Similar to Chitinase.
*Os12g0571000*	2.43	2.5	OsMIT1g, Metallothionein-like protein type 1.
*Os04g0486600*	-	-	OsGAPDH, Similar to Glyceraldehyde-3-phosphate dehydrogenase, cytosolic 3

### Gene ontology

**Figure 2:**
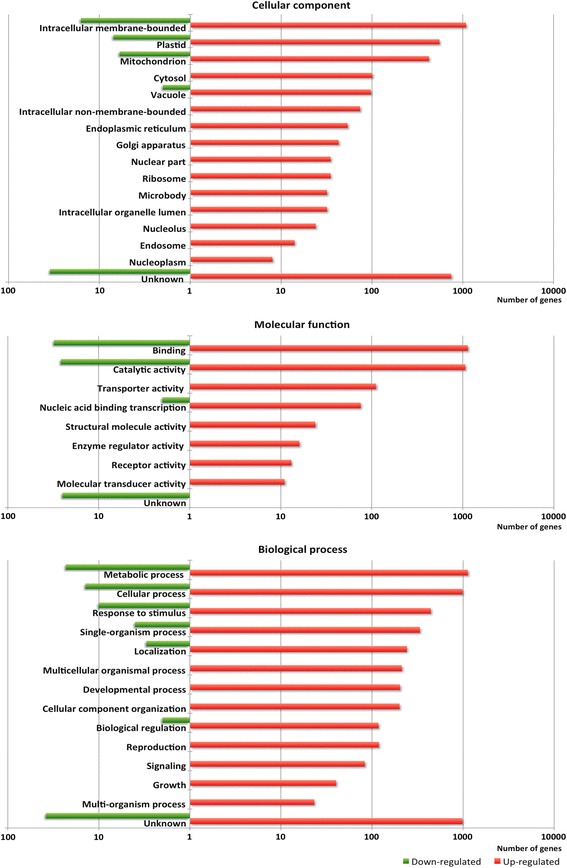
**Number of up-regulated and down-regulated genes in leaves of 18-day-old rice seedlings (** ***Oryza sativa*** **ssp.** ***japonica*** **cv. Nipponbare) after four days of iron excess exposure.** Gene ontology was generated by GO Slim with Biological Process and Molecular Function level 2 and Cellular Component level 6. A logarithmic scale was used for the number of genes.

The cellular component classification after GO analysis (Figure 2) shows the greatest number of gene products are located in an intracellular membrane bound organelle, and most up-regulated gene products are localized in plastids (558) or in mitochondria (428), followed by comparable numbers in the cytosol (101) and vacuole (97). The down-regulated genes have the same distribution, with the exception of the cytosol. Concerning molecular functions, the binding and catalytic activity are the most highly represented with, respectively, 1159 and 1085 genes for those which are up-regulated and 31 and 26 for down-regulated genes. For transporter activity, only up-regulated genes (111) are observed under iron excess. For biological processes, the most frequent are metabolic process with 1187 up-regulated genes which represent more than one-third of genes affected by iron excess, followed by cellular process (996) and response to stimulus (450). The majority of up-regulated genes in the metabolic pathway are involved in lipid metabolic process (184), carbohydrate metabolism (175), biosynthesis of secondary metabolites (113) and biosynthesis of plant hormones (60) (Additional file 2: Table S2). Among the down-regulated genes, methane metabolism (5 genes) is particularly affected. In biological processes, another function with a large number of up-regulated genes is transport with 239 up-regulated genes.

### Expression profile of genes

Among the 2525 differentially expressed genes, 1773 correspond to genes with known functions (1720 up-regulated and 53 down-regulated) and 752 are hypothetical genes or proteins (Additional file 1: Table S1). Thus, for the following analysis we concentrated on those with a known function. We compared these differentially expressed genes with genes or families of genes already described in the literature as encoding proteins involved in iron metabolism (*i.e.* transporters and enzymes involved in iron homeostasis) (Gross et al. 2003; Zheng et al. 2009; Ricachenevsky et al. 2010; Ricachenevsky et al. 2011; Kobayashi and Nishizawa 2012; Quinet et al. 2012; Victoria et al. 2012; Wang et al. 2013; Dabarni et al. 2013). In our study, we found 61 genes up-regulated in shoots in response to iron excess in common with the 392 candidate genes (Table 3). Among these, we found genes encoding proteins involved in iron uptake, distribution, redistribution and storage. These were classified into 3 classes according to their putative functions in iron homeostasis: (1) iron acquisition and mobilization (2) iron transport (3) regulatory mechanisms for iron responses.

**Table 3:** **List of genes found both in our experiments and which are known to be involved in iron homeostasis**

**Gene-ID**	**Description**	**log**_**2**_ **FC**
**Iron uptake**		
Os04g0578600	OsFRO2, ferric-chelate reductase/oxidase protein	1.40
Os02g0306401	OsNAAT1, Similar to Nicotianamine aminotransferase A	1.34
Os04g0659300	OsRMC, receptor like protein	3.04
Os06g0486800	OsFDH, Similar to Formate dehydrogenase, mitochondrial precursor	1.46
**Transport**		
Os01g0238700	OsYSL1	1.45
Os08g0280300	OsYSL17	1.78
Os09g0396900	OsVIT1, putative vacuolar iron/manganese transporter	1.47
Os01g0503400	OSNRAMP6	1.43
Os07g0232800	OsZIP8	1.53
Os06g0566300	OsZIP10	1.25
Os08g0467400	OsZIP14	1.87
Os01g0279400	OsZIFL2, Major facilitator superfamily antiporter	−1.82
Os11g0135900	OsZIFL7, Major facilitator superfamily protein	1.09
Os12g0133300	OsZIFL13, Similar to Carbohydrate transporter/sugar porter/transporter	1.13
Os03g0571900	OsPEZ1, phenolics efflux transporter	1.61
Os01g0684900	Multi antimicrobial extrusion protein MatE family protein	1.07
Os10g0345100	Multi antimicrobial extrusion protein MatE family protein	1.16
Os06g0495500	Multi antimicrobial extrusion protein MatE family protein	1.55
Os09g0440700	OsCOPT7, putative copper cation transporter	1.14
Os01g0304100	OsCCC2, putative cation:chloride co-transporter	1.29
**Homeostasis**		
Os03g0288000	OsMT1b, Similar to Metallothionein	1.12
Os05g0202800	OsMT3b, Similar to Metallothionein-like protein 3B	1.55
Os12g0567800	OsMT1f, Plant metallothionein, family 15 protein	2.01
Os12g0571000	OsMT1g, Metallothionein-like protein type 1	2.53
**Transcription factors**		
Os01g0816100	OsNAC4, Similar to NAC domain protein	1.60
Os01g0884300	OsNAC6, No apical meristem (NAM) protein domain containing protein	1.20
Os03g0339100	OsPRL1	1.01
Os03g0860100	OsEBP1 (ethylene responsive element binding protein encoding gene)	1.94
**Stress oxydatif**		
Os08g0561700	OsSOD4, OsCSD4, Similar to Superoxide dismutase	1.23
Os12g0613250	Similar to BTB/POZ; Superoxide dismutase, copper/zinc binding; NPH3	1.27
Os07g0665300	Similar to Superoxide dismutase	1.69
Os03g0285700	OsAPX1, APXa, OsAPx01, Similar to L-ascorbate peroxidase	1.20
Os10g0415300	OsGR3, GR3, Similar to Chloroplastic glutathione reductase	1.02
Os02g0280700	Similar to Iron/ascorbate-dependent oxidoreductase	1.42
Os07g0531400	Similar to Peroxidase 27 precursor (EC 1.11.1.7) (PRXR7)	1.20
Os11g0112200	Similar to Cationic peroxidase 1 precursor (EC 1.11.1.7) (PNPC1)	2.09
Os01g0327100	Haem peroxidase family protein	1.81
Os10g0527400	Similar to Tau class GST protein 3	1.54
Os10g0530900	Similar to Glutathione S-transferase GST 30 (EC 2.5.1.18)	1.85
**Hormonal regulation**		
Os03g0860100	OsEBP1 (ethylene responsive element binding protein encoding gene)	1.94
Os02g0201900	DNA/RNA helicase, C-terminal domain containing protein	1.54
Os04g0475600	2OG-Fe(II) oxygenase domain containing protein. (ethylene)	1.35
Os05g0178600	Similar to Auxin-responsive protein (Aux/IAA) (Fragment)	1.01
Os02g0643800	Auxin responsive SAUR protein family protein	1.02
Os09g0491740	Auxin efflux carrier domain containing protein	1.14
Os09g0554300	Auxin efflux carrier domain containing protein	1.20
Os10g0147400	Similar to Auxin influx carrier protein	1.25
Os12g0529300	Similar to Auxin-binding protein	1.51
Os04g0288100	Similar to Auxin-binding protein ABP20	1.76
Os07g0164900	Similar to ABA aldehyde oxidase	1.29
Os12g0555200	Similar to Probenazole-inducible protein PBZ1. Gibberellins	1.13
**Cytochrome P450**		
Os03g0760200	Cytochrome P450 family protein	1.71
Os02g0570700	Cytochrome P450 family protein	1.21
Os07g0418500	Similar to Cytochrome P450	2.77
Os07g0635500	Similar to Cytochrome P450	1.89
Os10g0515900	Cytochrome P450 family protein	1.26
**Senescence and stress marker**		
Os04g0650000	ORYZAIN, Similar to Oryzain alpha chain precursor (EC 3.4.22.-)	1.32
Os04g0670200	Similar to Oryzain beta chain precursor (EC 3.4.22.-)	1.80
Os02g0709800	OsGAP1. RabGAP/TBC domain containing protein	1.33
Os09g0532000	OsSGR, TonB box, conserved site domain containing protein	1.62
Os04g0600300	OsAOX1b, Homodimeric diiron-carboxylate protein, Cyanide-resistant respiration in mitochondria	1.79

Concerning genes involved in iron acquisition and mobilization, few of them were induced by iron excess in shoots. Transcription levels of only some genes involved in strategy I and II varied under iron excess. Among them, the ferric-chelate reductase oxidase gene, *OsFRO2* (*Os04g0578600*), involved in strategy I for the reduction of Fe^3+^ and also described as playing a role in shoots (Sperotto et al. 2010) was up-regulated under iron excess in our condition. Expression of genes implicated in strategy II, the nicotianamine aminotransferase, *OsNAAT1* (*Os02g0306401*) (Inoue et al. 2008), and a receptor like protein RMC, *OsRMC* (*Os04g0659300*), recently shown to be involved in regulation of iron acquisition in rice (Yang et al. 2013), were up-regulated under iron excess. We found genes coding for known or potential metal transporters. The *YSL* (*yellow stripe-like*) genes are known as components of strategy II and are believed to transport NA-metal chelates across plant cell membranes. Experimental evidence points to a role of the YSL proteins in the long-distance and intra-cellular transport of metals (Curie et al. 2009; Ishimaru et al. 2010). Of the 18 rice genes that belong to the *OsYSL* family (Koike et al. 2004), two were induced in our conditions (*OsYSL1, Os01g0238700* and *OsYSL17, Os08g0280300*).

Vacuolar sequestration is an important mechanism in regulating iron homeostasis, and could serve as a safe iron storage strategy. Several genes have been shown to regulate iron trafficking between the cytosol and vacuoles, among which *OsVIT1* (*Os09g0396900*), localized to the vacuolar membrane and able to transport Fe^2+^ across the tonoplast into the vacuole (Zhang et al. 2012), was induced under our conditions. Among the 8 genes in the family of natural resistance-associated macrophage protein (NRAMP) metal cation transporters shown to be involved in metal uptake and transport in plants (Belouchi et al. 1997; Narayanan et al. 2007; Takahashi et al. 2011), only *OsNRAMP6* (*Os01g0503400*) was up-regulated in our conditions. It has been shown that zinc transporters may play important roles in iron homeostasis in plants (Ricachenevsky et al. 2011). In the *ZIP* (*Zinc Iron Permease*) family genes, known to participate in divalent metal transport in plant (Guerinot et al. 2000; Lee et al. 2010), twelve homologs of the transporter OsIRT1 are present in the rice genome (Ishimaru et al. 2005). Among them, *OsZIP8* (*Os07g0232800*), *OsZIP10* (*Os06g0566300*) and *OsZIP14* (*Os08g0467400*) were up-regulated in iron toxicity. For the *Zinc-induced facilitator-like* (*ZIFL*) family genes, among the 13 genes of this family in rice genome (Ricachenevsky et al. 2011), *OsZIFL2* (*Os01g0279400*) was down-regulated and *OsZIFL7* (*Os11g0135900*) and *OsZIFL13* (*Os12g0133300*) up-regulated in our conditions.

The *Phenolic Efflux Zero1* (*OsPEZ1*, *Os03g0571900*) gene, shown to load protocatechuic acid (PCA) and caffeic acid into the rice xylem facilitating remobilization of precipitated apoplasmic iron inside the plant (Ishimaru et al. 2011), was induced under iron excess. Three up-regulated genes (*Os01g0684900, Os10g0345100, Os06g0495500*), belonging to the family of citrate transporters (Multi antimicrobial extrusion protein MATE family protein), were observed under iron excess. Some members of this family were found to be involved in the efficient translocation of iron from roots to shoots in rice (Yokosho et al. 2009).

Concerning the regulatory mechanisms for iron responses, we found four up-regulated genes (*Os03g0288000, Os05g0202800, Os12g0567800, Os12g0571000*) coding for metallothioneins (MTs) that have already been shown to have a significant role in maintaining intracellular metal homeostasis, metal detoxification and protection against intracellular oxidative damage (Zhou et al. 2006; Nath et al. 2014). MTs participate in controlling the concentration of “free” metals and reactive oxygen species that would activate defenses, *e.g. via* the MAPK cascade. These responses would help to regain cellular oxidant and metal homeostasis (Polle and Schützendübel 2003).

A large number of up-regulated genes (130) encoding transcription factors (TFs) was observed, mostly zinc finger (46 up-regulated genes) and WRKY (21 up-regulated genes) transcription factors. Other families of TFs encoded by up-regulated genes were bHLH (helix-loop-helix) (9 genes), ERFs (5 genes) and MYB (8 genes). Among the transcription factors, four were previously reported to be associated with iron response (Table 3): *OsNAC4* (*Os01g0816100*) and *OsEBP1* (*Os03g0860100*) by Zheng et al. (2009) and Wu et al. (2011), *OsNAC6* (*Os01g0884300*) by Nakashima et al. (2007) and Todaka et al. (2012), and *OsPRL1* (*Os03g0339100*) by Duc et al. (2009) and (Dabarni et al. 2013).

Antioxidant/scavenger enzymes are involved in detoxification of reactive oxygen species (ROS) produced under iron stress toxicity (Becker and Asch 2005). Most of the genes involved in oxidative stress and detoxification of ROS were up-regulated in response to iron excess. Among the up-regulated genes, we found 9 genes coding for glutathione S-transferases (GSTs) which catalyze the conjugation of the reduced form of glutathione (GSH) to xenobiotic substrates for the purpose of detoxification in the cells. We also found genes encoding antioxidant enzymes, for example three superoxide dismutase (SOD) genes, two L-ascorbate peroxidase (APX) genes, seven genes encoding proteins similar to peroxidase (PRX) and one encoding chloroplastic glutathione reductase (GR).

Sixty up-regulated genes are involved in the biosynthesis of plant hormones (Additional file 3: Table S3). Plant hormones are implicated in the regulation of assimilate metabolism and growth. Furthermore, some of them, like abscisic acid (ABA), auxin and ethylene play a major role in controlling iron homeostasis in plants (Chen et al. 2010; Lingam et al. 2011; Wu et al. 2011). Among the up-regulated genes in our condition, we found genes implicated in hormone responses (Table 3) like the ABA signaling pathway (1 gene), auxin response (7 genes), ethylene response (3 genes, among them *OsEBP1*) and gibberellin response (1 gene).

Among genes involved in oxygen and electron transfers, the Cytochrome P450 family was represented by 44 up-regulated genes, most of which have a molecular function of monooxygenase activity or iron ion binding. Cytochrome P450 monooxygenases (P450s) play an essential role in the synthesis of lignin, pigments, defense compounds, fatty acids, hormones and signaling molecules in all plant species (Schuler 1996; Werck-Reichhart et al. 2002; Nielsen and Moller 2005, Pan et al. 2009). Recently, Quinet et al. (2012) found 11 up-regulated cytochrome P450 genes in the roots of *indica* rice (cv. Kong Pao) after three days of iron exposure. Among these genes, six (*Os08g0105700*, *Os03g0760200, Os02g0570700, Os07g0418500, Os07g0635500* and *Os10g0515900*) are also up-regulated in our conditions (Table 4), suggesting that these enzymes are important for iron homeostasis.

**Table 4:** **List of genes common to our experiments and Quinet et al.** 2012

**Gene-ID**	**log**_**2**_ **FC**	**Function in RAP-DB**	**Function in Quinet et al.** 2012
**Carbohydrate metabolism**			
Os03g0169000	2.30	Similar to predicted protein	Ribulose-5-phosphate-3-epimerase
Os04g0275100	1.12	Serine/threonine protein kinase domain containing protein	Wall-associated kinase
Os05g0366600	1.07	Similar to Hydroxyisourate hydrolase	Beta-glucosidase
Os07g0539900	1.40	Similar to Beta-1,3-glucanase-like protein	O-Glycosyl hydrolase
Os07g0538000	1.21	Similar to hydrolase, hydrolyzing O-glycosyl compounds	1-3,1-4-beta-glucanase
Os04g0459500	1.01	Similar to GADPH (383 AA) (Fragment)	Glyceraldehyde-3-phosphate dehydrogenase
Os10g0416500	1.10	Similar to Chitinase 1 precursor (EC 3.2.1.14)	Chitinase
**Metabolism of secondary products**			
Os03g0122300	1.09	Similar to Oxidoreductase, 2OG-Fe oxygenase family protein	Flavanone 3-hydroxylase-like protein
Os02g0218700	1.58	Similar to Allene oxide synthase (EC 4.2.1.92)	Allene oxide synthase
**Metabolism of toxins**			
Os10g0530900	1.85	Similar to Glutathione S-transferase GST 30 (EC 2.5.1.18)	Glutathione S-transferase (OsGSTU6)
**Oxygene and electron transfer**			
Os04g0600300	1.79	Homodimeric diiron-carboxylate protein,	Alternative oxidase
Os08g0105700	2.91	Similar to Bx2-like protein	Cytochrome P450
Os03g0760200	1.71	Cytochrome P450 family protein	Cytochrome P450
Os02g0570700	1.21	Cytochrome P450 family protein	Cytochrome P450
Os07g0418500	2.77	Similar to Cytochrome P450	Cytochrome P450
Os07g0635500	1.89	Similar to Cytochrome P450	Cytochrome P450
Os10g0515900	1.26	Cytochrome P450 family protein	Cytochrome P450
Os02g0218700	1.58	Similar to Allene oxide synthase (EC 4.2.1.92)	Allene oxide synthase
Os06g0549900	1.90	FAD linked oxidase, N-terminal domain containing protein	Reticulilne oxidase-like protein
Os05g0529700	1.44	Heat shock protein DnaJ family protein	DNAJ heat shock protein
**Oxidative stress and detoxification**			
Os07g0531400	1.20	Similar to Peroxidase 27 precursor (EC 1.11.1.7) (PRXR7)	Peroxidase
Os11g0112200	2.09	Similar to Cationic peroxidase 1 precursor (EC 1.11.1.7)	Cationic peroxidase
Os01g0327100	1.81	Haem peroxidase family protein	Peroxidase
Os10g0527400	1.54	Similar to Tau class GST protein 3	Glutathione S-transferase (OsGSTU3)
Os10g0530900	1.85	Similar to Glutathione S-transferase GST 30 (EC 2.5.1.18)	Glutathione S-transferase (OsGSTU6)
Os04g0447700	1.50	Similar to Polyketide reductase	NADPH-dependent oxidoreductase
Os03g0700700	1.73	Similar to Lipoxygenase	Lipoxygenase
**Iron homeostasis**			
Os09g0396900	1.47	OsVIT1,Protein of unknown function DUF125	CCC1, iron transporter
Os04g0538400	1.24	Similar to Nodulin 21 (N-21)	Vacuolar iron transporter
**Hormonal regulation**			
Os12g0555200	1.13	Similar to Probenazole-inducible protein PBZ1	Probenazole-inductible protein PBZ1
Os02g0201900	1.54	DNA/RNA helicase, C-terminal domain containing protein	Ethylene response factor (ERF)
Os04g0275100	1.12	Serine/threonine protein kinase-related domain containing protein	Wall-associated kinase
Os02g0218700	1.58	Similar to Allene oxide synthase (EC 4.2.1.92)	Allene oxide synthase

It is interesting to note that some differentially expressed genes are members of gene families already described as related to stress response or senescence. For example, oryzain-alpha and OsGAP1 were already characterized as related to iron excess in rice (Ricachenevsky et al. 2010). Oryzain-alpha (*Os04g0650000*), which is a cysteine proteinase found to be up-regulated by various stresses in rice leaves and which has been suggested to be involved in the final steps of leaf senescence (Fu et al. 2007), is up-regulated in our conditions, as is oryzain-beta (*Os04g0670200*). The C2 domain protein OsGAP1 (*Os02g0709800*) is also up-regulated. Among the other genes known as senescence and stress markers, we observed over-expression of *OsSGR* (*Os09g0532000*) (Park et al. 2007) and *OsAOX1b* (*Os04g0600300*) which codes for, an alternative oxidase involved in reactive oxygen species (ROS) scavenging (Saika et al. 2002; Feng et al. 2013).

### Occurrence of cis-Regulatory Elements (CREs) in putative promoters of up-regulated genes

**Figure 3:**
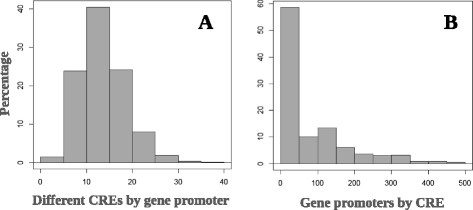
**Histograms of significant (P < 0,05) occurrences of different CREs in each gene promoter and number of gene promoters in which each CRE occurs. A)** Histogram with a percentage of different CREs by gene promoter of up-regulated genes in leaves of 18-day-old rice seedlings (*Oryza sativa* ssp. *japonica* cv. Nipponbare) after four days of iron excess exposure; **B)** Histogram representing the percentage of gene promoters in which each CRE occurs.

For the identification of known plant regulatory promoter elements, 1 kbp upstream from transcription start sites of up-regulated genes were analyzed for potential consensus sequences using the 469 CREs experimentally validated in the literature (Mangeon et al. 2010; Tsutsui et al. 2011) and in the PLACE Database. We found 338 predicted CREs with a significant occurrence (p ≤ 0.05). The number of different predicted CREs in 1 kb upstream regions ranged from zero to 40 (Figure 3A). Individual CREs were found in zero to 495 different putative promoter regions (Figure 3B).

Among the most represented CREs, ACGTABOX, GT1CORE, ARFAT, RYREPEATGMGY2, TATABOX3 and AMYBOX1 are each present in more than 400 up-regulated genes. Some of them have been identified in many plant genes regulated by diverse environmental and physiological conditions (Green et al. 1988; Huang et al. 1990; Lelievre et al. 1992; Foster et al. 1994). Interestingly, ARFAT has been shown to be an auxin-responsive element (Ulmasov et al. 1999). Additionally, among the 29 ABA responsive CREs (Table 5), we identified 23 that have at least one significant occurrence in the 1 kb region upstream from 665 up-regulated genes in response to iron toxicity (this represents *ca*. 30% of up-regulated genes). The most frequent CREs were ABREOSRAB21, ABRERATCAL and ACGTABREMOTIFA2OSEM that are present in 226, 272 and 293 putative gene promoters, respectively. The 665 up-regulated genes include kinases, transcription factors and genes involved in ROS detoxification. The list of up-regulated genes that present at least one occurrence of an ABA-responsive CRE is provided in Additional file 4: Table S4.

**Table 5:** **Number of promoters of up-regulated genes and LTRs of LTR-retrotransposons where ABA responsive CREs are significantly present (P < 0.05)**

**ABA responsive CREs**	**Number of gene promoters**	**Number of LTR-retrotranposons**
ABAREG2	0	0
ABASEED1	0	0
ABRE2HVA22	0	3
ABRE3OSRAB16	0	0
ABRECE3HVA1	0	0
ABRECE3ZMRAB28	0	4
ABRE2HVA1	1	1
ABRE3HVA1	1	0
ABRE3HVA22	1	0
ABADESI2	2	1
ABREDISTBBNNAPA	2	0
ABREMOTIFIOSRAB16B	2	0
ABRETAEM	2	0
ABREBNNAPA	3	0
ABREBZMRAB28	3	0
ABREMOTIFIIIOSRAB16B	3	3
ABREAZMRAB28	6	0
ABRECE1HVA22	8	1
ABADESI1	9	0
ABREA2HVA1	16	0
ABREMOTIFAOSOSEM	29	2
ACGTABREMOTIFAOSOSEM	29	2
ABREATRD22	38	48
ABREZMRAB28	67	103
ABREATCONSENSUS	118	110
ABRELATERD1	154	65
ABREOSRAB21	226	167
ABRERATCAL	272	128
ACGTABREMOTIFA2OSEM	293	152

The occurrences of CREs in the 1 kb upstream regions were separated into two groups: the putative complex regulation group in which CRE occurrence is greater than or equal to the average occurrence in all genes plus two standard deviations (*i.e.* more than 24 different CREs in the 1 kb upstream region); and the putative simple regulation group, when CRE occurrence is smaller than or equal to the average occurrence in all genes minus two standard deviations (*i.e.* less than 4 different CREs in the 1 kb upstream region). Examples of simple and complex regulation are the up-regulated gene *Os06g0127800,* a GAI-RGA-SCR (GRAS) family protein involved in a Brassinosteroid signaling, with 35 different CREs with a significant occurrence and thus classified as a gene with putative complex regulation, and the up-regulated gene *Os03g0815200,* that has a function similar to methylenetetrahydrofolate reductase (EC 1.5.1.20), with only one CRE in the 1 kb upstream region, classified as having putative simple regulation.

**Figure 4:**
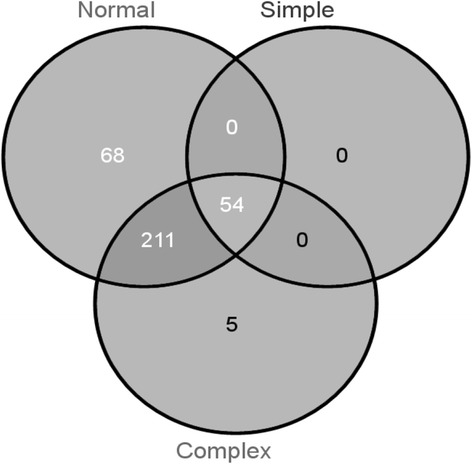
**Venn diagram showing numbers of different CREs present in each group (complex, simple and normal regulation).** Complex regulation was considered when the CRE occurrence was greater than or equal to the average of occurrences in all up-regulated genes plus two standard deviations. The group of simple regulation was considered when the CRE occurrences was smaller than or equal to the average of occurrences in all up- regulated genes minus two standard deviations. CREs that occurs in the gene promoter in the interval between complex and simple regulation are considered to have normal regulation.

We found 102 genes in the complex regulation group and 19 in the simple regulation group, and 54 and 270 different CREs in groups of genes with putative simple and complex regulation, respectively. The 54 CREs observed in the putative simple regulation group were also found in the other group. In contrast, 68 CREs were only present in normal genes and 5 only in the putative complex regulation groups (Figure 4). The latter 5 are: C1MOTIFZMBZ2, GBOXSORBCS1, RYREPEAT4, ABRETAEM and ACGTSEED3.

### Rice transposable elements

We completed the gene transcriptomic survey with an analysis of LTR-retrotransposons. We used a microarray that contained oligomers corresponding to previously described elements (Chaparro et al. 2007) to observe the transcriptional activity of all LTR-retrotransposons in the rice genome. More recently, El Baidouri and Panaud (2013) performed a new classification of rice LTR-retrotransposons, defining 369 families and 3623 loci harbouring complete LTR-retrotransposons in the rice genome. We used this latest data for our analysis, retaining only those that had 100% identity and which were unique in the genome among the differentially expressed oligomers.

In the presence of an excess of iron, differential expression was observed for 158 LTR-retrotransposon families among the 369 existing families in rice. When we looked in detail, the stress modified the transcription of 37% (1344 loci/3623 loci present in rice genome) of complete LTR-retrotransposons, among which 95.4% were up-regulated and 4.6% down-regulated. We previously showed in another stress (a rice mutant line derived from an *in vitro* callus culture) that 8 LTR-retrotransposon families were activated at the transpositional level in rice (Sabot et al. 2011). Interestingly, the majority of these families (seven out of eight) were found to be differentially expressed in our study: *BAJIE, osr10, osr37, rn363, rn216, RIRE2* and *RIRE3.*

In addition, we explored if transposable elements can significantly alter the expression of adjacent genes. In the rice genome, LTR-retrotransposons can be present within the region of 3 kb upstream or downstream genes. In our microarray, we do not have the complete flanking region for all genes. However, among the differentially expressed LTR-retrotransposons, 10% are located in the neighborhood of genes. In fact, 57 are located within 3 kb upstream from genes which are differentially expressed under iron stress, while 65 are located in the 3 kb downstream from other differentially expressed genes. We checked whether these elements have an impact on the expression of the adjacent gene. No co-transcription events between genes and LTR-retrotransposons could be detected by RT-PCR (data not shown), suggesting that none of these elements presented direct read-through transcription of adjacent genes. We therefore looked for CREs in the complete LTRs of LTR-retrotransposons differentially expressed in our conditions, because the LTR contains the promoter region of the element. We found 1247 LTRs that contain at least one CRE and, among the 469 analysed CREs, 279 were found with a significant occurrence (p ≤ 0.05). The most representative was ARFAT, present in more than 470 up-regulated LTR-retrotransposons. Comparing these results with those for genes, we found that, among the 8 most highly-represented CREs in the putative promoters of up-regulated genes, 5 are also found in the LTRs of LTR-up-regulated retrotransposons (TATABOX3, ARFAT, GT1CORE, HEXMOTIFTAH3H4, LTRE1HVBLT49). Interestingly, when we compared the distribution of TATABOX3 in all LTRs (differentially expressed or not) the overall distribution of this CRE is significant in LTRs of LTR-retrotransposons up-regulated under iron excess (test chi^2^ p < 0.001).

Additionally, among the 29 ABA responsive CREs (Table 5), we identified 15 that have at least one significant occurrence in LTR of up-regulated LTR-retrotransposons in response to iron toxicity. The most frequent CREs were ABREOSRAB21, ABRERATCAL and ACGTABREMOTIFA2OSEM that are present in 167, 128 and 152 LTR of LTR-retrotransposons, respectively. This is exactly the same pattern as the one we found for putative promoters of up-regulated genes. Our analysis shows that genes and LTR-retrotransposons up-regulated under iron toxicity present the same pattern of CREs.

## Discussion and Conclusions

Previous studies on the Nipponbare cultivar led to contradictory results. Wan et al. (2003), using a nutritive solution with 250 mg L^−1^ of Fe^2+^, described it as being tolerant to iron excess, as the leaf bronzing index was not significantly different to Suakoko8 (the iron toxicity tolerant control cultivar) after 28 days exposure to iron excess. However, more recently, Engel et al. (2012) studied different rice genotypes, among which Nipponbare and Kong Pao cultivars, for response after 4, 6 and 8-week-old plants to an Fe pulse of 1,500 mg L^−1^ Fe^2+^ for 6 days. Nipponbare was considered susceptible because they observed that 8-week-old plants of Nipponbare showed a leaf-bronzing score significantly higher than the genotypes considered tolerant. Furthermore, taking into account the above-ground biomass accumulation, no significant differences were found between Nipponbare and tolerant genotypes. The Kong Pao cultivar was classified as tolerant to iron toxicity. The Nipponbare used in this experiment comes from the same stock used for the IRGSP, and has performed in several experiments as medium tolerant to tolerant to iron excess (Costa de Oliveira, pers.commun.).

We compared our observations with those of Quinet et al. (2012), who studied gene expression in 2-week Kong Pao (*indica* rice) plants in response to 125 mg L^−1^ FeSO_4_ for 3 days (short-term) and 3 weeks (long-term). We find that only a few genes in *japonica* rice are the same as those responsive to iron toxicity in *indica* rice (Table 4). None of the down-regulated genes are the same as in *indica* rice shoots in short-term response. Quinet et al. (2012) find 135 down and 95 up-regulated genes in shoots in the short-term response, whereas we found more than 10 times as many up-regulated genes. This difference could be due to the fact that in our experiment we performed a global analysis with a microarray that contained probes corresponding to all genes of the *japonica* rice genome and all complete LTR-retrotransposons.

Although we did not find exactly the same genes, it is interesting to note that we often found genes belonging to the same family or involved in the same metabolic pathway. In fact, a large number of genes up-regulated in our experiment are involved in carbohydrate and secondary metabolism, oxidative stress and detoxification of ROS, iron homeostasis (YSL, NRAMP, ZIP, metallothionein) and hormonal response (ABA signaling pathway, auxin, ethylene), as found by Quinet et al. 2012. In addition, these authors observed no difference in leaf iron accumulation after iron excess exposure, in contrast to our experiment in *japonica* rice where we obtained twice as much iron as in the rice control. The absorbed iron would be stored in ferritins and immobilized by chelation (Briat et al. 1999; Zancani et al. 2004). However, no changes in gene expression were observed for iron storage proteins like ferritins in our study. Similarly, Quinet et al. (2012) did not observe any change in expression of ferritin genes in leaves during long or short-term responses. In their experiments, ferritins were only up-regulated in roots in short-term responses. Studies in which *OsFER1* and *OsFER2* were observed to be over-expressed in rice (Stein et al. 2009b) used higher iron concentrations than in our conditions.

In a recent study, Sperotto et al. (2010) showed that in eight rice cultivars, in the same conditions, the expression pattern of 25 metal-related genes varied among the 8 cultivars and revealed contrasting levels of iron in seeds between cultivars. Subsequently, Banerjee and Chandel (2011) also observed the expression of 43 metal homeostasis related genes in 12 rice cultivars and showed significant genotypic variation in their levels of expression. We find very few genes in common in the transcriptomic profiling with the short-term response to iron excess in *indica* and *japonica* rice. The differences could be inherent to responses of subspecies, or could be associated with the Kong Pao tolerance (as reported by Engel et al. 2012). We speculate that the differences observed between our experiments and Quinet et al. are due to the rice genotypes and the experimental protocols used in the two studies.

In this study, a large number of transporters have been identified and the GO analysis revealed that transport activity is activated in response to iron excess. These proteins may have an important role in transport and sequestration of iron. However, much still remains to be elucidated on intracellular metal transport involving vacuoles, chloroplasts and mitochondria, although some transporters have been identified with specific iron translocation roles for these compartments (Ishimaru et al. 2006; Lee et al. 2010; Ishimaru et al. 2011; Yuan et al. 2011; Zhang et al. 2012). Despite these advances, the coordinated function of different transporters that have a role in iron homeostasis is not fully understood. It will be interesting to characterize the different genes highlighted in our study.

Our study highlights the importance of oxidative stress and detoxification of ROS. In fact, oxidative stress is a harmful process with potentially deleterious effects on plant metabolism that have to be avoided by mobilization of antioxidant defense. However, Kuzniak (2010) pointed out that ROS are key regulators of biological processes in plant biology. It is now generally accepted that the effects of ROS result from direct or indirect responses of sensing systems involving antioxidants, rather than from oxidative damage to bio-molecules*.* For example, glutathione metabolism is a pathway that is involved in the antioxidative system of plants, reduced glutathione (GSH) playing a central role by scavenging ROS (Ranieri et al. 2005). It is generally considered that GSH content positively correlates with metal stress (Tausz et al. 2004) and participates in plant defense against oxidative stress and toxicity generated from heavy metals (Marrs 1996). The stimulation by iron of superoxide dismutase, ascorbate peroxidase and gluthatione reductase, as observed in our conditions, has already been highlighted in rice (Fang et al. 2001 and Majerus et al. 2007). Another group of up-regulated target genes identified in this work is composed of heavy metal detoxification genes, such as cytochrome P450, metallothioneins, and MATE family of transporters that can help to reduce ROS production in mitochondria. Most up-regulated genes encode proteins that act in mitochondria or plastids, organelles that are involved in iron homeostasis and ROS production.

Very recently, Nakashima et al. (2014) performed promoter analysis of drought-responsive genes in rice plants among which *OsNAC6,* that we found up-regulated under iron excess. Transient assays using promoters indicated that AREB/ABF (ABA-responsive element-binding protein) transcription factors enhanced expression of this gene and GUS assays revealed that the *OsNAC6* promoter was induced by drought, high salinity and ABA treatment (Nakashima et al. 2014). In our study, the *in silico* analysis revealed a large number of up-regulated genes (*ca.* 30% of up-regulated genes) possessing ABA cis-regulatory elements. These results suggest that ABA may have an important role in response to iron toxicity.

The analysis of CREs allowed us to identify common CREs between promoters of genes and the LTRs of LTR-retrotransposons up-regulated in our condition, which are sequences (CREs) potentially involved in driving expression in iron excess. Though these sequence patterns require experimental validation, our current findings may open new avenues for studying the regulation of gene and LTR-retrotransposon expression in iron excess. It has already been demonstrated that some LTRs harbor cis-regulatory signals that confer responsiveness to various external stimuli and play a role in reactivation of transposition (Takeda et al. 1999). In addition, LTRs can serve as alternative promoters or enhancers to regulate genes as far away as 40 kb (Pi et al. 2007; Romanish et al. 2007). Our analysis emphasizes the role of LTR-retrotransposons in the evolutionary and environmental adaptation of plants. Furthermore transposable elements are maintained silent by epigenetic mechanisms in normal conditions (Feschotte et al. 2002). It would be interesting to further analyze whether the silencing mechanisms are alleviated in our iron toxicity conditions.

In this paper, our microarray allowed identification of a large number of up-regulated genes and activated LTR-retrotransposons and highlighted pathways involved in response to iron excess in *japonica* rice. We aimed to identify the CREs associated with this expression data using *in silico* prediction tools. The up-regulated genes present putative cis-regulatory elements in their promoter region indicating that many transcription factors may modulate the expression of these genes, and that these genes are probably multi-stress responsive. In addition, some CREs were common to genes and LTR-retrotransposons. These motifs could be used to design experimental verification of regulatory elements and the identification of transcription factors that regulate gene expression under iron excess. In fact, gene regulation is a crucial step to allow plant adaptation in response of fluctuating environments. Plants induce or repress various genes related to iron homeostasis in response to iron excess. Although our results shed new light on the response to iron excess, much remains to be discovered on the efficient strategies of stress adaptation.

## Methods

### Plant material

Pre-germinated seedlings of rice (cv. Nipponbare) with *ca*. 1 cm of rootlet were transferred to pots containing a complete nutrient solution as described by Camargo and Oliveira (1981): 4 μM Ca(NO3)_2_; 2 μM MgSO_4_; 4 μM KNO_3_; 0.435 μM (NH4)_2_SO_4_; 0.5 μM KH_2_PO_4_; 2 μM MnSO_4_; 0.3 μM CuSO_4_; 0.8 μM ZnSO_4_; 30 μM NaCl; 10 μM Fe-EDTA; 0.10 μM Na_2_MoSO_4_; 10 μM H_3_BO_3_ and were grown in these conditions for 14 days. The nutrient solution was changed every week, the pH was adjusted to 5.5. Subsequently, for iron excess treatment, the plants were transferred to pots containing nutrient solution supplemented with 7 mM of Fe^2+^, at pH 4.5 for four days. Control treatment plants were also changed on the 14th day to a complete nutrient solution at pH 4.5. The experiment consisted of three replicates in a completely random design, each replicate consisting of 100 seedlings. During the experiment, the photoperiod was 16 hours and photon flux density of 25 μmol m^−2^ s^−1^. On the 18th day, the leaves were collected and stored at −80°C until extraction of total RNA.

### RNA extraction and cDNA synthesis

Total RNA was extracted from a 2 g sample of each replicate according to the protocol extraction TRIzol™ Reagent (Invitrogen, Carlsbad, CA, USA) and purified with the RNeasy plant mini kit (Qiagen, Courtaboeuf, France) according to the manufacturer’s protocol. The quality of RNA was verified by electrophoresis on an agarose gel (1%) and RNAs assayed by spectrophotometry at a wavelength of 260 nm. cDNA synthesis was performed from mRNA according to the protocol Synthesis of Double-Stranded cDNA (NimbleGen) using the SuperScript™ Double-Stranded cDNA Synthesis Kit (Invitrogen), using oligo-dT(15) primers (Promega). Subsequently, the quantity of cDNA was measured by spectrophotometry, retaining samples with a UV absorbance ratio > 1.7 at A260/A280 and > 1.5 at A260/A230.

### Microarray hybridizations and designs

Six hybridizations were performed, three with samples from control conditions that grew in nutritive solution and three with samples treated with 390 mg L^−1^ of Fe^2+^. We designed an oligomer microarray, similar to one which was described previously (Picault et al. 2009). The oligomer microarray was produced by NimbleGen™ (Madison, WI, USA) and is composed of about 385,000 60mer probes selected for their GC content, Tm and number of cycles needed to synthesize the oligomers. This chip contains 90,000 probes representing 45,000 genes (2 probes per gene) of rice *Oryza sativa* ssp. j*aponica* and 290,000 probes representing copies of LTR retrotranposons. Gene probes were designed at the 3′ end of the genes to allow detection by hybridization with potentially partial reverse transcriptase products. In contrast, LTR-retrotransposons are represented throughout their length at the rate of one oligonucleotide for each 500 bp.

When it was possible, oligonucleotides were designed to be unique in the genome (*i.e.* locus specific), to avoid problems of oligonucleotide redundancy on the chip. Hybrids with up to three mismatches were considered to be stable enough to withstand the conditions of washing after the chip hybridization during hybridization between a cDNA and an oligonucleotide. The oligonucleotides are therefore considered to be locus specific when they do not match elsewhere and have less than four mismatches.

### Data verification, spatial effect correction and normalization

Data verification was performed by comparing the average, standard deviation and quantiles of each sample, in order to highlight the samples which present excessively different contributions. This verification was performed with the R statistical software (R Development Core Team 2012) using the boxplot and summary command. This enabled us to identify any sample with inappropriate hybridizations.

The first step consists in reducing the space bias effect for each hybridization. Space biases are due mainly to poor washing after hybridization, or a misallocation of signal, manifested by a gradient of intensity. These biases were corrected with the script SpatialSmooth of NMPP software (NimbleGen Microarray Data Processing Pipeline - NMPP) (Wang et al. 2006) through a global distance-weighted smoothing algorithm. After the spatial smooth, quantile normalization adjusts values in a microarray experiment to improve consistency and reduce technical biases and variations between hybridizations. Normalization between hybridizations was performed with the QuantileNorm script of NMPP software (Wang et al. 2006). Quantile normalizations force the arrays of a set of experiments to have absolutely identical distribution. It is based on the assumption that the RNA populations hybridized to the arrays should be the same. The global normalization adjusts each condition to the same baseline (the median) in order to allow the hybridizations of the different conditions to be comparable. Analysis of variance with a false discovery rate adjustment method was realized (Benjamini and Hochberg 1995).

The results of different treatment comparison were calculated as Log_2_-fold change. The oligonucleotides selected were those which presented a two-fold increase or decrease in expression, *i.e.*, a log-fold change smaller or equal to −1 for down-regulation, and greater or equal to 1 for up-regulation. Oligonucleotides displaying P-value ≤0.05 for the statistical test were selected.

### Oligonucleotide annotation and gene ontology

A local alignment (BLAST) (Altschul et al. 1990) for all oligonucleotides differentially expressed on the chip by considering a log_2_-fold-change ≥ 1 and ≤ −1 was carried out using the database IRGSP 1.0. Gene ontology and metabolic pathway classifications were performed using the software Blast2GO (Conesa, et al. 2005) with Gene Ontology (Ashburner et al. 2000) and KEGG (Kyoto Encyclopedia of Genes and Genomes) (Kanehisa et al. 2012) databases, respectively. The Venn diagram was performed with Venny tool (Oliveros 2007).

### Experimental validation of microarray by RT-qPCR

Chip validation was performed by RT-qPCR (reverse transcriptase quantitative PCR) using primer pairs for 16 up-regulated and 3 down-regulated genes. Primer design was performed in Primer Express® software (Applied Biosystems, California, United States). Total RNA was digested with DNase I™. The RNA quality was checked by agarose gel electrophoresis and RNA quantity by measuring the absorbance at 260 nm. cDNA first-strand synthesis was performed with a SuperScript™ First-Strand Synthesis System for RT-PCR (Invitrogen) from 2 μg of RNA. RT-qPCR was performed in an Applied Biosystems 7500 Fast Real-Time PCR System using a SYBR Green detection system (Applied Biosystems, California, United States). The ΔΔCt relative quantification method (Livak and Schmittgen 2001), in which the expression data of the target genes were normalized with the level of expression of GAPDH (Glyceraldehyde 3-phosphate dehydrogenase) as reference gene, were used, with three technical replicates. The RT-qPCR experiment was performed according to MIQE guidelines (Bustin et al. 2009). Amplification was done with Taq Platinum (Invitrogen) with the following program: 50°C for 30s, 95°C for 10s; 40 cycles of 95°C for 30s, 60°C for 1 min, and 72°C for 1 min and a final elongation at 72°C for 5 min. Pearson’s correlation was performed between the data obtained by microarray and RT-qPCR for each gene validated, using the Hmisc Package v. 2.14.0 of the R statistical software (R Development Core Team 2012).

### Quantification of micronutrients in the tissue of rice shoots

The content of micronutrients such as copper (Cu), zinc (Zn), manganese (Mn) and iron (Fe) was determined according to the methodology described by Tedesco et al. (1995) using three replicates for each condition. The data obtained in atomic absorption spectrometry (Thermo Scientific) was calculated based on a standard curve for each element. The non-normality and heterogeneity of the data was checked and an ANOVA F-test was performed using the R statistical software (R Development Core Team 2012).

### Cis-Regulatory Element (CREs) pattern search in putative promoters of up-regulated genes and LTRs of retrotransposons

The putative promoters (1.0 Kbp upstream portions of start site of transcription) of up-regulated genes (2,457 genes considering log_2_FC ≥ 1) and the LTRs of LTR-retrotransposons were obtained from RAP-DB database. PLACE – a database of plant *cis*-acting regulatory DNA elements (http://www.dna.affrc.go.jp/PLACE/index.html) (Higo et al. 1999) and PLANTICS (Pegoraro et al. 2013) were employed to search for information (ID, consensus sequences, TFs related) about reported *cis-*acting regulatory elements (CREs). A Z score for the occurrences for each of 469 CREs in the 2,457 putative promoters of up-regulated genes was calculated in order to verify if the probability of the results found was not random. A cutoff of 0.05 (or 5%) was used to eliminate false positives (Rombauts et al. 2003).

## Additional files

Additional file 1: Table S1. List of 2525 genes differentially expressed in leaves of 18-day-old rice seedlings (*Oryza sativa* ssp. *japonica* cv. Nipponbare) after four days of iron excess exposure: 2.457 were up-regulated and 68 were down-regulated: locus ID, fold-change (log2FC) and description of genes. Additional file 2: Table S2. Number of up and down-regulated genes in leaves of 18-day-old rice seedlings (*Oryza sativa* ssp. *japonica* cv. Nipponbare) after four days of iron excess exposure, and its respective metabolic pathway generated by Kegg (Kyoto Encyclopedia of Genes and Genomes). Additional file 3: Table S3. Number of up-regulated genes involved in biosynthesis of plant hormones in leaves of 18-day-old rice seedlings (*Oryza sativa* ssp. *japonica* cv. Nipponbare) after four days of iron excess exposure, generated by Kegg (Kyoto Encyclopedia of Genes and Genomes). Additional file 4: Table S4. Number of significant occurrences of ABA responsive *cis*-regulatory elements in promoter (1 kbp upstream) of up-regulated genes in leaves of 18-day-old rice seedlings (*Oryza sativa* ssp. *japonica* cv. Nipponbare) after four days of iron excess exposure: locus ID, fold-change (log2FC) and description of 20-ABADESI1, 21- ABADESI2, 25-ABRE2HVA1, 27-ABRE3HVA1, 28-ABRE3HVA22, 30-ABREA2HVA1, 31-ABREATCONSENSUS, 32-ABREATRD22, 33-ABREAZMRAB28,- 34- BREBNNAPA, 35-ABREBZMRAB28, 36-ABRECE1HVA22, 39-ABREDISTBBNNAPA, 40-ABRELATERD1, 41-ABREMOTIFAOSOSEM, 42-BREMOTIFIIIOSRAB16B, 43-ABREMOTIFIOSRAB16B, 44-ABREOSRAB21, 45-ABRERATCAL, 46-ABRETAEM, 47-ABREZMRAB28, 50-ACGTABREMOTIFA2OSEM, and 51-ACGTABREMOTIFAOSOSEM. NBS – number of binding sites, NDCRE (number of different cis-regulatory elements).
